# The role of rk39 serologic test in the diagnosis of visceral leishmaniasis in a Tertiary Hospital, Northern Ethiopia

**DOI:** 10.1186/s13104-017-2490-3

**Published:** 2017-04-26

**Authors:** Yazezew Kebede Kiros, Bethlhem Feleke Regassa

**Affiliations:** 1 Department of Internal Medicine, College of Heath Sciences, Mekelle University, P.O.Box 1711, Mekelle, Ethiopia; 2 Ayder Referral Hospital, Mekelle, Ethiopia

**Keywords:** rK39, Visceral leishmaniasis, Ayder Referral Hospital, Northern Ethiopia, Mekelle University

## Abstract

**Background:**

The study is done in Ayder Referral Hospital in Northern Ethiopia. Ethiopia is one of the countries where visceral leishmaniasis (VL) is endemic. Diagnosis of VL in Ethiopia is primarily based on rK39 immunochromatographic (rk39-ICT) strip. This test has been shown to have variable sensitivity and specificity in different countries. Hence the objective of the study is to determine the sensitivity and specificity of rk39-ICT in the diagnosis of VL in our set up. The study participants were VL suspected patients admitted to the hospital. A cross sectional study design was used. The study was conducted from January 14, 2013 to June 26, 2015. The rK39-ICT strip used was the InBios brand. Ethical clearance was obtained from the IRB of the college and written consent was obtained from the individual patients.

**Results:**

A total of 62 VL suspects were involved in the study. The mean age was 26.3 years (SD = 6.94 years) with a median age of 25.5 years. Sixty-one (98.4%) of the patients was males. The rK39-ICT was positive in 50 (80.6%) of the patients. Splenic aspiration was positive in 44 (71%) of the patients. In 37 (59.7%) of the patients both rK39 and splenic aspiration were positive. Thirteen (21%) of the patients had positive rK39 but negative splenic aspiration. Five (8.1%) of the patients had both negative rK39 and splenic aspiration however seven (11.3%) of the patients had rk39 negative but splenic aspiration positive. The sensitivity, specificity, positive predictive value and the negative predictive value of rK39-ICT, taking splenic aspiration as a gold standard test, is 84.1% (95% CI 69.9–93.4%), 27.8% (95% CI 9.7–53.5%), 74.0% (95% CI 60–85.4%) and 41.7% (95% CI 15.2–72.3%) respectively.

**Conclusion:**

Sensitivity of rK39-ICT is low and its specificity is poor in our set up. Significant number of patients with confirmed VL did not have travel history to endemic areas. We recommend that the rK39-ICT needs improvement for clinical use in our set up and case definition for visceral leishmaniasis in Ethiopia needs to be revisited.

## Background

The term leishmaniasis refers to a group of vector-borne diseases caused by obligate intracellular protozoan parasites of the genus Leishmania. Natural transmission of the parasite occurs primarily via the bite of infected female sandflies [1]. Leishmaniasis occurs in different clinical forms but VL is the most severe form of the disease, and if it is left untreated, it is usually fatal. Leishmaniasis is prevalent in more than 98 countries worldwide. However 90% of VL occurs mainly in Bangladesh, Brazil, Ethiopia, India, Nepal, South Sudan, and Sudan [2]. In Ethiopia, VL is found mainly in the lowlands of northwest, central, south and southwestern Ethiopia. It is estimated that about 5000 cases occur annually in Ethiopia [3]. Northwestern part of Tigray is one of the endemic areas in Ethiopia. VL in Ethiopia is caused by *Leishmania donovani* and the vectors are *Phlebotomus martini*, *Phlebotomus Celiae* and *Phlebotomus orientalis* [3].

Laboratory diagnosis of VL includes detection of Leishmania by direct microscopic or culture in clinical samples, detection of antigen or specific antibodies and detection of the DNA of the parasite. Definitive diagnosis of VL requires demonstration of the parasite from a tissue aspirate such as the spleen, bone marrow, or lymph node. However, as this is quite an invasive procedure, with its inherent complication, it is not widely practiced in most health institutions in Ethiopia; hence, VL diagnosis is primarily based on rK39-ICT and occasionally DAT serologic tests [3]. However, serologic tests have important limitations. One important limitation is that it does not differentiate exposure from active VL [4]. The second is its cross reactivity with other common infections like tuberculosis and malaria [1]. The co-infection of VL with HIV also affects the serology based diagnosis as this category of people may not mount adequate antibodies [4].

Despite these limitations, multiple validity studies of the rk39-ICT conducted in different countries [4–7] and a meta analysis study [8] showed good sensitivity and specificity. However, the sensitivity and specificity of rK39-ICT serologic test are quite variable in different settings [3]. One Study done in Ethiopia showed a higher sensitivity and specificity for rK39-ICT of more than 90% [9] however another study done in Ethiopia showed low sensitivity of about 72% [10]. In a study done in Sudan the sensitivity of rK39-ICT was 92% but the specificity was as low as 59% [11]. In another Sudanese study the sensitivity of rK39-ICT in clinically suspected cases is 81% and the specificity was 97% [12] indicating the inconsistency of the test strip in different set ups. Another multicentre study found that the sensitivity of rK39-ICT is low in East Africa compared with other regions and recommended that it has to be monitored carefully in its clinical application [3, 13]. Therefore, the objective of our study is to assess the value of rK39-ICT serologic test in the diagnosis of VL suspected patients in Ayder Referral Hospital.

## Methods

The aim of the study is to determine the sensitivity and specificity of the rK39-ICT for the diagnosis of VL. The study was conducted in Ayder Referral Hospital found in Mekelle city, Northern Ethiopia. The hospital is a 500 bedded university teaching hospital and serves as a referral hospital for an estimated 8 million people of the northern part Ethiopia. The study period was from January 14, 2013 to June 26, 2015. A cross sectional study design was used. Patients were recruited continuously throughout the study period. All clinically suspected VL cases for whom both splenic aspiration and rk39-ICT serologic tests were done were included in our study. The exclusion criteria was a patient in whom either rk39-ICT and/or splenic aspiration was not done.

Fresh peripheral whole blood was drawn by respective ward nurses for rK39-ICT determination. The test was performed in the central laboratory of the hospital. The test kit used was the InBios rK39-ICT strip kit. Splenic aspiration was performed by the respective treating physicians in the wards and the microscopic interpretation was done by a pathologist. Both the pathologist and the laboratory technicians were not aware that the patient samples were included in a study. All were also blinded to the clinical status of the patients. The specimens collected for the analysis were collected for clinical diagnosis and further management. The investigators were not involved in any clinical decision making what so ever but they interviewed the patients further for additional socioeconomic and clinical data. Symptoms were systematically checked and recorded during visit by the investigators. Physical finding were also crosschecked by one of the investigators (who is an internal medicine specialist by profession). The turnaround time for rK39-ICT result was same day but for splenic aspiration was 3 days. Ethical clearance was obtained from the IRB of the College of Health Sciences at Mekelle University. Written consent for willingness to participate in the study was obtained from the patients.

**Fig. 1:**
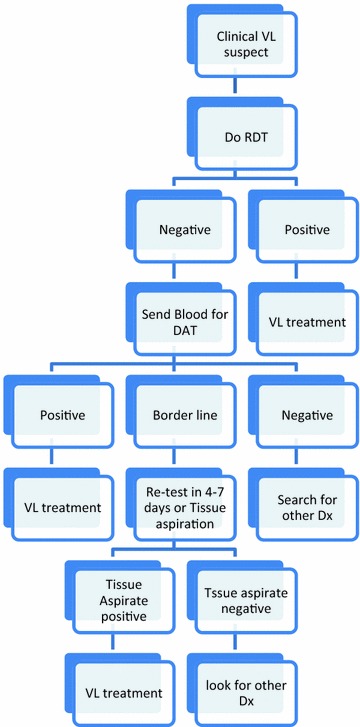
Diagnostic algorithm for VL in Ethiopia, Ministry of health of Ethiopia, 2013

The VL case definition in Ethiopia is a person who presents with fever for more than 2 weeks and an enlarged spleen (splenomegaly) and/or enlarged lymph nodes (lymphadenopathy), or either loss of weight, anemia or leucopenia while living in a known VL endemic area or having travelled to an endemic area [3]. The diagnostic algorithm for VL in Ethiopia is depicted in Fig. 1 below.

Initially data were entered into an excel spread sheet, Microsoft office excel 2007. It was later exported to SPSS 16.0 version for analysis. Mean, median, standard deviation, percentages, sensitivities, specificities and positive and negative predictive values were calculated. © 1993–2016 MedCalc statistical Software bvba was used to calculate sensitivity, specificity and the positive and negative predictive values.

## Results

A total of 62 patients were analyzed in the study. The mean age of patients is 26.3 years (SD = 6.94 years) with a median age of 25.5 years. Sixty-one (98.4%) of the patients was males. Ten (16.1%) of the patients did not have travel history to the known VL endemic areas. Ten (16.1%) of the patients did not fulfill the case definition for VL. Fever, loss of appetite, loss of weight, splenomegaly and lymphadenopathy were seen in 90.3, 92, and 95%, 96.8 and 8.1% respectively. (see Table 1).

**Table 1:** Socio demographic and clinical data of VL suspected patients in Ayder Referral Hospital, Northern Ethiopia

Variable	Frequency (%)
Sex	
Male	61 (98.4)
Female	1 (1.6)
Age	
15–20	14 (22.6)
21–25	15 (24.2)
26–30	19 (30.6)
31–35	8 (12.9)
36–40	3 (3.8)
41–50	3 (4.8)
Residence	
Urban	11 (17.7)
Rural	51 (82.3)
Occupation	
Daily labourers	27 (43.5)
Farmers	31 (50.0)
Civil servants	4 (6.5)
Travel history	52 (83.9)
Fever	56 (90.3)
Loss of appetite	57 (91.9)
Weight loss	59 (95.2)
Splenomegaly	60 (96.8)
Lymphadenopathy	5 (8.1)

The mean white blood cell count (WBC), hemoglobin (Hgb) and platelets counts are 2300 cell/dl, 8.3 g/dl and 123,500 cells/dl respectively. rK39-ICT test was positive in 50 (80.6%) of the patients. Splenic aspiration was positive in 44 (71%) of the patients. In 37 (59.7%) of the patients, both rK39-ICT and splenic aspiration were positive. Thirteen (21%) of the patients had positive rK39-ICT but negative splenic aspiration. Five (8.1%) of the patients had both negative rK39 and splenic aspiration; however, seven (11.3%) of the patients had rK39-ICT negative but splenic aspiration positive. (see Table 2). The sensitivity, specificity, positive predictive value and the negative predictive value of rK39-ICT, taking splenic aspiration as a gold standard test, is 84.1% (95% CI 69.9–93.4%), 27.8% (95% CI 9.7–53.5%), 74.0% (95% CI 60–85.4%) and 41.7% (95% CI 15.2–72.3%) respectively.

**Table 2:** Results of splenic aspiration and rK39-ICT in VL suspected patients in Ayder Referral Hospital, Northern Ethiopia

	Splenic aspirate parasitologic result		Total
	Negative	Positive	
rK39 ICT strip test			
Negative	5	7	12
Positive	13	37	50
Total	18	44	62

## Discussion

In our study 43 out of 44 (97.7%) bacteriologically confirmed VL cases are males. More cases are reported in males compared to females from other endemic countries [14]. Specifically in our set up, this might be due to increased exposure of males as they are engaged in big commercial farms found in the endemic areas in Ethiopia. But it might also be due to the limited access of women to health care. However the gap is so wide that further study is warranted. Ten (16.1%) parasitologically confirmed cases of VL did not have travel history to known VL endemic area or have never resided in such places. This is in contrast to the case definition for VL in Ethiopia which puts travel history as mandatory to fulfill the case definition [3]. The sensitivity of rK39-ICT in our study is 84.1% (95% CI 69.9–93.4%). This is comparable to a large meta analysis of RDTs for the diagnosis of VL by Boelaert M et al. which showed a sensitivity of 85.3% for rK39-ICT [13]. There is also a multicentre study involving Ethiopia, Sudan, Kenyia and India by Boelaert et al. which showed sensitivity of below 80% in the three African countries [15] and another study done in Ethiopia by ter Horst et al. showed sensitivity of 84% [16]. However, our finding is lower compared to studies conducted in Ethiopia by Cañavate et al. which showed sensitivity of 94.3 and 91.4% [9] for two different brands and also other studies in India [5, 7], Brazil [6] and Sudan [11, 12], which showed sensitivities of 100% and 98, 90,92 and 90% respectively. In one large meta-analysis, the combined sensitivity of rK39 was 93.9% [8]. Our finding is higher compared to a study conducted in Ethiopia which showed a sensitivity of 71.7% [10]. In general, our finding is comparable to studies done in Ethiopia and other East African countries but lower than studies done elsewhere. Previous studies also demonstrated that sensitivity in East Africa is generally low [14, 15] and some experts even advise the use of rK39 in East Africa to be closely monitored [15]. It is to be noted that the Ethiopian National VL guideline recommends for a diagnostic test to be highly sensitive (>95%) and specific to be clinically useful [3].

The specificity of rK39-ICT in our study is 27.8% (95% CI 9.7–53.5%). Our finding is significantly lower compared to studies conducted in Ethiopia which showed specificities of 70% [15], 82.4% [10], 90.6% [8] and 98.5 and 94% [9] for two different brands, 98% [16] and in India [5, 7], Sudan [13] and Brazil [6] which showed specificities of 98% and 89, 99 and 100% respectively though one study from Sudan showed a lower specificity of 59% [11]. Though our finding is lower, specificity of rK39 in general is quite variable in different set ups. For example, in one meta-analysis study, specificity ranged from 66.8 to 97.9% which is quite wide [8]. This might be due to the fact that patients can develop antibodies after exposure without developing active clinical disease. The second reason might be that there is cross reactivity [1] with other epidemiologically common infections like tuberculosis and malaria in our set up.

The positive predictive value of rK39-ICT in our study is 74.0% (95% CI 60–85.4%) which is lower than the study done in Sudan 81% [11]. This shows that rK39-ICT positivity does not necessarily indicate the presence of active VL in significant portion of individuals. The negative predictive value is 41.7% (95% CI 15.2–72.3%). This indicates that rK39-ICT negative test does not rule out the presence of VL in a patient. The same conclusion has been drawn by other studies [4]. Although our study has come up with a different finding which will have significant implication in the diagnosis of VL patients in our set up it has also an important limitation due to the limited sample size and the fact that HIV status of our patients were not known.

## Conclusion

Sensitivity of rK39-ICT is low and its specificity is poor in our set up. Significant number of patients with confirmed VL did not have travel history to endemic areas. We recommend that the rK39-ICT needs improvement for clinical use in our set up and case definition for visceral leishmaniasis in Ethiopia needs to be revisited.

## Abbreviations

Hgb: hemoglobin
RDT: rapid diagnostic test
rK39-ICT: rK39 immunochromatographic test
VL: visceral leishmaniasis
WBC: white blood cell count
